# Interfacial chemical bond and internal electric field modulated Z-scheme S_v_-ZnIn_2_S_4_/MoSe_2_ photocatalyst for efficient hydrogen evolution

**DOI:** 10.1038/s41467-021-24511-z

**Published:** 2021-07-05

**Authors:** Xuehua Wang, Xianghu Wang, Jianfeng Huang, Shaoxiang Li, Alan Meng, Zhenjiang Li

**Affiliations:** 1 College of Materials Science and Engineering, Qingdao University of Science and Technology, Qingdao, Shandong P. R. China; 2 Key Laboratory of Optic-electric Sensing and Analytical Chemistry for Life Science, MOE. College of Chemistry and Molecular Engineering, Qingdao University of Science and Technology, Qingdao, Shandong P. R. China; 3 School of Material Science and Engineering, International S&T Cooperation Foundation of Shaanxi Province, Xi’an Key Laboratory of Green Manufacture of Ceramic Materials, Shaanxi University of Science and Technology, Xi’an, China; 4 Shandong Engineering Technology Research Center for Advanced Coating, Qingdao University of Science and Technology, Qingdao, P. R. China; 5 College of Sino-German Science and Technology, Qingdao University of Science and Technology, Qingdao, Shandong P. R. China

**Keywords:** Artificial photosynthesis, Photocatalysis, Two-dimensional materials, Characterization and analytical techniques, Design, synthesis and processing

## Abstract

Construction of Z-scheme heterostructure is of great significance for realizing efficient photocatalytic water splitting. However, the conscious modulation of Z-scheme charge transfer is still a great challenge. Herein, interfacial Mo-S bond and internal electric field modulated Z-scheme heterostructure composed by sulfur vacancies-rich ZnIn_2_S_4_ and MoSe_2_ was rationally fabricated for efficient photocatalytic hydrogen evolution. Systematic investigations reveal that Mo-S bond and internal electric field induce the Z-scheme charge transfer mechanism as confirmed by the surface photovoltage spectra, DMPO spin-trapping electron paramagnetic resonance spectra and density functional theory calculations. Under the intense synergy among the Mo-S bond, internal electric field and S-vacancies, the optimized photocatalyst exhibits high hydrogen evolution rate of 63.21 mmol∙g^−1^·h^−1^ with an apparent quantum yield of 76.48% at 420 nm monochromatic light, which is about 18.8-fold of the pristine ZIS. This work affords a useful inspiration on consciously modulating Z-scheme charge transfer by atomic-level interface control and internal electric field to signally promote the photocatalytic performance.

## Abstract (web summary)

The construction of Z-scheme heterostructures is of great significance for realizing efficient photocatalytic water splitting. Here, the authors report an interfacial chemical bond and internal electric field modulated Z-Scheme S_v_-ZnIn_2_S_4_/MoSe_2_ photocatalyst for efficient hydrogen evolution.

## Introduction

With the rapid development of the industrial society, the energy crisis and environmental pollution issues are getting more and more serious. Therefore, finding an alternative energy source is of great significance for the long-term development of human society. Hydrogen (H_2_) has long been considered as an excellent candidate to substitute the fossil fuel, due to its advantages of clean, renewable, high energy density, and transportability^1–3^. However, at present, the low efficiency, high energy consumption, and environmentally hazardous H_2_ production technology seriously restrict the commercial application of hydrogen energy. By comparison, photocatalytic water splitting can tactfully convert the sustainable solar energy to H_2_ energy without discharging any pollutant during the whole process, thus has been considered as a sustainable and promising technique^1,4,5^.

In the past few years, metal chalcogenide semiconductor photocatalyst, such as ZnS, CdS, PbS, ZnIn_2_S_4_, have absorbed extensively attention due to the favorable visible-light response ability^6–8^. ZnIn_2_S_4_ is a typical ternary layered metal chalcogenide semiconductor with adjustable band gap of 2.06~2.85 eV, besides, the conduction band is about −1.21 eV, suggesting the intense reducing capacity of the photogenerated electrons^9^. In addition to the suitable band structure, ZnIn_2_S_4_ also possess the prominent photo-stability, environmental and human friendliness in comparison to CdS and PbS^10^. Whereas, the photocatalytic property of the single ZnIn_2_S_4_ is unsatisfying because of the serious carrier recombination. For pursuing the higher photocatalytic activity of ZnIn_2_S_4_, researchers have thrown tremendous efforts, including phase and morphology regulating, elements doping, cocatalyst-loading, defect engineering, and heterojunction constructing^11–15^. Among these strategies, defect engineering and heterojunction constructing are the two-effective means. In photocatalytic field, introducing anion vacancies in semiconductor can not only enhance the light absorption ability of the pristine semiconductor, but also introduce mid gap states in the band gap, which can serve as effective electron “traps” accelerating the separation efficiency of photocarriers^16^. Nevertheless, the excessive defects in photocatalyst can also act as the recombination sites of photocarriers, thus deteriorating the photocatalytic performance^17^. Therefore, regulating the defect in an appropriate concentration would ensure the high activity and stability of photocatalyst^18^. In addition, as known from the reported literatures, the only defect introduction is not enough for realizing efficient photocatalytic property.

Heterojunction constructed by coupling different materials with diverse energy level structure is another effective means to improve photocatalytic performance^19–23^. In recent years, Z-scheme heterostructure, especially the direct Z-scheme heterostructure, has become one of the most effective strategy for obtaining high-efficient photocatalyst ^22,23^. For example, Huang et al. reported a HxMoO_3_@ZnIn_2_S_4_ direct Z-scheme photocatalyst for efficient hydrogen production. The results demonstrates that the H_x_MoO_3_@ZnIn_2_S_4_ presents a 10.5 times higher H_2_-production activity (5.9 mmol·g^−1^·h^−1^) than pristine ZnIn_2_S_4_^24^. To fabricate the Z-scheme heterostructure, the primary premise is the matching band structure, in which the conduction band of one semiconductor should locate as close to the valence band of another semiconductor as possible. It is reported that the conduction band potential of MoSe_2_ (about −0.45 eV^25^) is lower than the conduction band of ZnIn_2_S_4_, but very close to its valence band (0.99 eV^9^), which suggests that the photogenerated electrons in the conduction band of MoSe_2_ are likely to recombine with the photogenerated holes in the valence band of ZnIn_2_S_4_ following Z-scheme pathway. However, as known from the current literatures, MoSe_2_ can only play the role of cocatalyst in ZnIn_2_S_4_/MoSe_2_ instead of realizing Z-scheme charge transfer^26,27^. The question is that there is no direct and intimate interfacial connection between MoSe_2_ and ZnIn_2_S_4_. The poor interfacial contact is like erecting a “wall” between the two semiconductors, seriously preventing the trajection of charge flow. Therefore, the formation of intimate interface contact became the hinge to Z-scheme photocatalyst fabrication.

Recently, defect-induced heterostructure construction have opened thought for assembling the heterostructure with specific atomic-level interfacial contact^22^. Its basic principle lies on that the defective sites with abundant coordinative unsaturation atoms and delocalize local electrons can act as the anchoring sites for other semiconductors to form a unique heterostructure contact interface with chemical bond connection^28^. The interfacial chemical bond can act as specific “bridge” accelerating charge transfer between semiconductors. In addition to the intimate interface combination, internal electric field also emerging as a viable strategy to promote Z-scheme charge transfer^29^. Under the effect of internal electric field, the photogenerated electrons in the conduction band of one semiconductor with lower Fermi level could directionally transfer to the valence band of another semiconductor with higher Fermi level, thus realizing the Z-scheme charge transfer^30^. Inspired by the above considerations, an efficient Z-scheme photocatalyst can be obtained through establishing intimate interfacial chemical bond connection between two semiconductors with specific band structure and Fermi level. Up to now, however, the interfacial bonding and internal electric field are always considered separately, the jointly modulation and their synergy effect on photocatalytic performance still remains a challenging task.

**Fig. 1:**
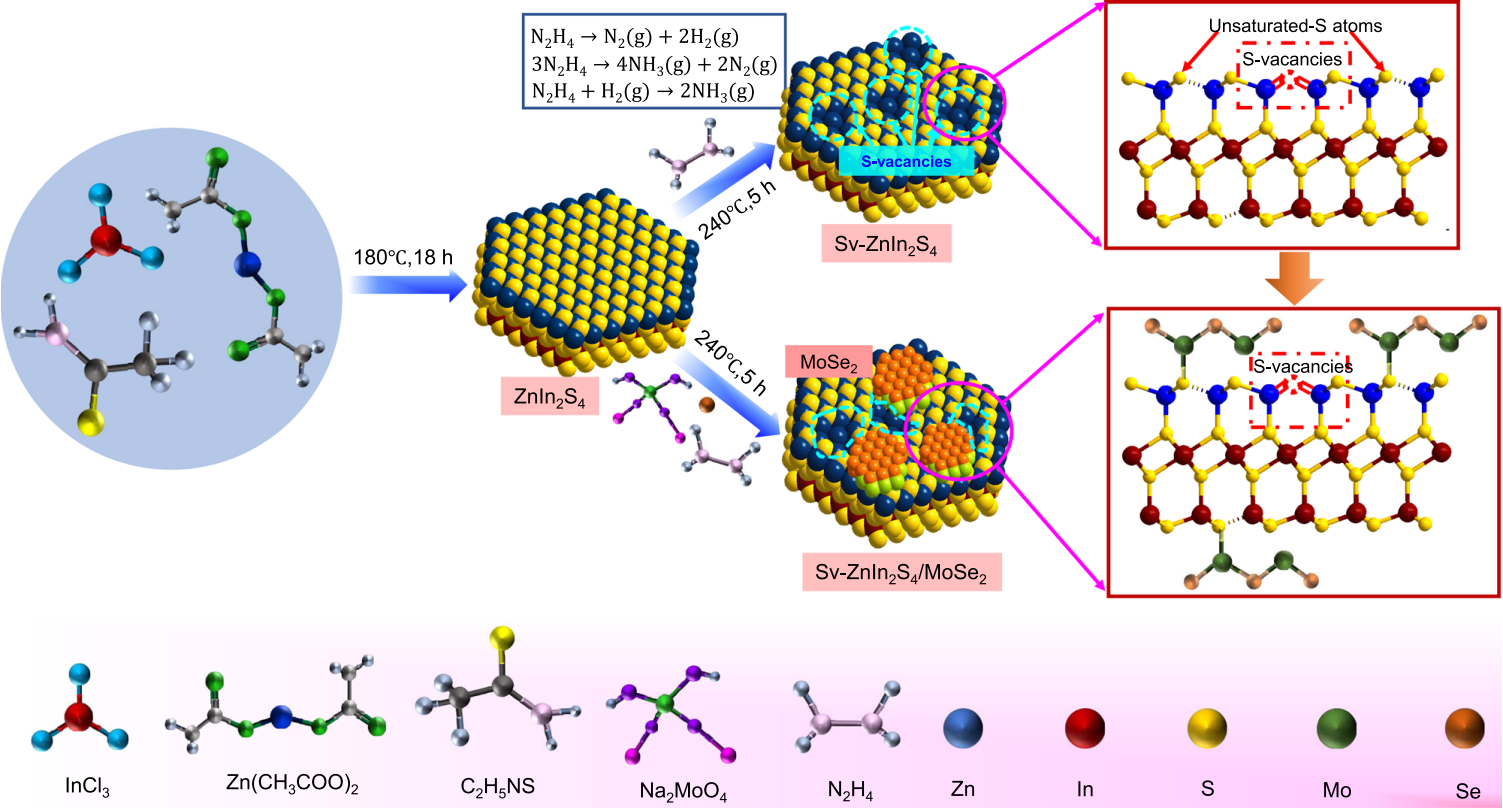
Synthesis process. Schematic presentation of the synthetic route of S_v_-ZnIn_2_S_4_ and S_v_-ZnIn_2_S_4_/MoSe_2_ heterostructure.

Herein, taking S vacancies-rich ZnIn_2_S_4_ (S_v_-ZIS) and MoSe_2_ as model material, through a defect-induced heterostructure constructing strategy, an interfacial Mo-S bond and internal electric field modulated Z-scheme S_v_-ZIS/MoSe_2_ photocatalyst was fabricated. The addition of hydrazine monohydrate (N_2_H_4_ ∙ H_2_O) provides pivotal prerequisite for the formation of S vacancies and coordinative unsaturation S atoms, where the S vacancies can enhance light absorption and facilitate photocarriers separation, while the abundant coordinative unsaturation S atoms can serve as anchoring sites for Mo atoms, thus contributing the formation of Mo-S bond and the in-situ growth of MoSe_2_ on the surface of S_v_-ZIS (as showing in Fig. 1). During photocatalytic reaction, the internal electric field induced by the different work function between S_v_-ZIS and MoSe_2_ provide intense driving force steering the photogenerated electrons on the conduction band of MoSe_2_ transfer to the valence band of S_v_-ZIS, that’s the Z-scheme mechanism. Meanwhile, the interfacial Mo-S bond afford the fast pathways for charge transfer from MoSe_2_ to S_v_-ZIS, thus accelerating the Z-scheme charge transfer process. This work provides a constructive reference for atomic-level interfacial and internal electric field regulating Z-scheme heterostructure for efficient photocatalytic reaction.

## Results and discussion

### Characterizations of as-prepared photocatalysts

**Fig. 2:**
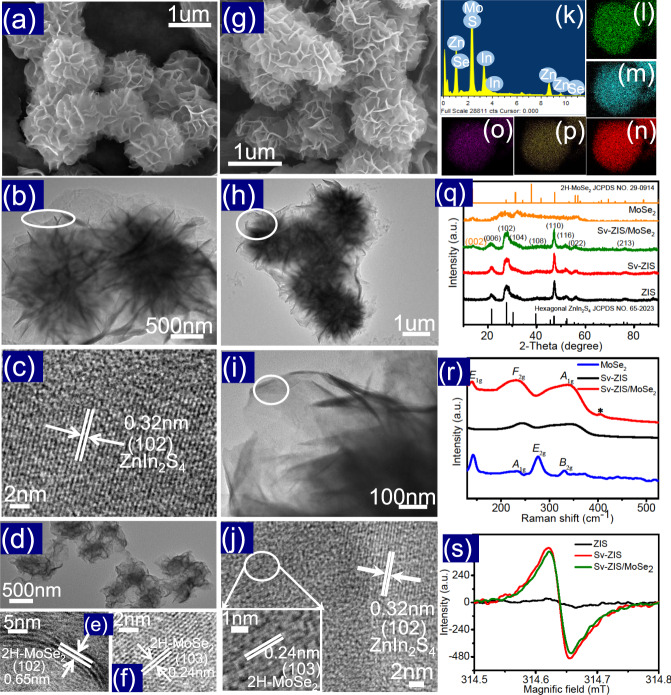
Morphology and composition characterizations. **a–c** SEM, TEM, and HRTEM pictures of ZIS, **d–f** TEM and HRTEM images of MoSe_2_, **g–j** SEM, TEM, and HRTEM images of S_v_-ZIS/MoSe_2_, **k–p** EDS and elements mapping of Zn, In, S, Mo, and Se in S_v_-ZIS/MoSe_2_, **q** XRD patterns of ZIS, S_v_-ZIS, MoSe_2_ and S_v_-ZIS/MoSe_2_, **r** Raman spectra of S_v_-ZIS, MoSe_2_ and S_v_-ZIS/MoSe_2_, and **s** EPR spectra of ZIS, S_v_-ZIS and S_v_-ZIS/MoSe_2_.

The morphology and microstructure of the as-synthesized ZIS, MoSe_2_ and S_v_-ZIS/MoSe_2_ (the optimized sample) were analyzed by the SEM, TEM and HRTEM characterizations. As observed in Fig. 2a, the basic morphology of ZIS is flower-like hierarchical microsphere composed by plenty of intersecting nanoflakes, which benefits to the exposure of active surface. The TEM image in Fig. 2b further reveals the hierarchical microsphere of ZIS assembled by nanoflakes. Furtherly, as shown in the HRTEM image in Fig. 2c, the clear lattice stripes with interplanar spacing (d) of 0.32 nm can be well indexed to the (102) lattice plane of hexagonal ZnIn_2_S_4_ (JCPDS:65-2023)^9^. Figures S1–S2 are the elements mapping and EDS spectrum of ZIS, it can be clearly seen the evenly distributed Zn, In and S elements, and the atomic ratio of Zn/In/S can be calculated to be about 1.00/1.85/4.13 (as listed in Table S1), very close to the stoichiometric ratio in ZnIn_2_S_4_. Figure S3 presents the SEM, TEM and element mapping images of the S_v_-ZIS. It is found that the S_v_-ZIS appears the identical morphology and structure with ZIS, suggesting that the N_2_H_4_ ∙ H_2_O-assisted hydrothermal treatment cannot destroy the flower-like microsphere structure of ZIS. The atomic ratio of Zn/In/S in S_v_-ZIS sample is ~1.00/1.92/3.35 (as displayed in Table S2), the distinctly deficient of S atom compared to that in ZIS confirms the existence of abundant S vacancies in ZIS. Figure 2d is the TEM picture of MoSe_2_, which manifests the nanosheet feature. The HRTEM image (Fig. 2e and f) present the d-spacing of 0.65 and 0.24 nm, assigning to the (002) and (103) lattice planes of 2H-MoSe_2_ (JCPDS: 29-0914), respectively^31^. Figure 2g is the SEM image of S_v_-ZIS/MoSe_2_, which exhibits almost the same morphology with ZIS, moreover, the ZIS and MoSe_2_ in the S_v_-ZIS/MoSe_2_ structure are undistinguishable, indicating that the MoSe_2_ was grown on the surface of ZIS intimately to form a 2D/2D contact, and the introduction of MoSe_2_ can hardly affect the hierarchical microsphere morphology of ZIS. The TEM image displaying in Fig. 2h and i further reveal the hierarchical flower-like microsphere structure of S_v_-ZIS/MoSe_2_, which could lead to the enhanced light absorption by the multilevel reflection and scattering of the incident light^32^. Furthermore, the HRTEM picture displaying in Fig. 2j shows the different lattice stripes with d value of 0.32 and 0.24 nm, respectively, which can be indexed to the (102) crystal face of hexagonal ZnIn_2_S_4_ (JCPDS:65-2023) and the (103) lattice planes of 2H-MoSe_2_ (JCPDS: 29-0914), respectively. The HRTEM results indicate that MoSe_2_ are directly grown and attach on the ZIS nanosheets substrate. Figure 2k-p is the EDS spectra and element mapping of S_v_-ZIS/MoSe_2_, as displayed, the distribution of Zn, In, S elements are dense and uniform, meanwhile, the Mo and Se elements are relatively sparse but still evenly distributed. From the EDS spectrum, the mass ratio of MoSe_2_ to ZIS can be calculated to be about 4.8% (as presented in Table S4), which is very close to the ratio of the added raw materials. What’s more, the atomic ratio of Zn/In/S in S_v_-ZIS/MoSe_2_ was determined to be 1.00/1.83/3.25, indicating that there is still a mass of S vacancies inside S_v_-ZIS/MoSe_2_.

The ZIS, S_v_-ZIS, MoSe_2_ and S_v_-ZIS/MoSe_2_ were further characterized by X-ray diffraction (XRD) to determine the phase composition. As displayed in Fig. 2q, the XRD pattern of MoSe_2_ matches well with 2H-MoSe_2_ (JCPDS:29-0914)^31^. Meanwhile, ZIS displays the distinct peaks at 21.6°, 27.7°, 30.4°, 39.8°, 47.2°, 52.4°, 55.6° and 76.4°, which can be severally indexed to the (006), (102), (104), (108), (110), (116), (022) and (213) crystal planes of hexagonal ZnIn_2_S_4_ (JCPDS:65-2023)^9^. It is worth noting that the S_v_-ZIS sample shows almost the same XRD pattern with ZIS, indicating that the introduction of S vacancies can hardly affect the size and crystal structure of ZIS. Moreover, in the XRD patterns of S_v_-ZIS/MoSe_2_, in addition to the peaks of hexagonal ZIS, a new peak at about 13.7° can be well assigned to the (002) crystal face of MoSe_2_, reconfirming the successful synthesis of S_v_-ZIS/MoSe_2_ composite.

To further characterize the chemical structures of the as-synthesized photocatalyst, the Raman spectra were carried out (shown in Fig. 2r). As observed in the Raman spectra of MoSe_2_, the peaks located at 235.4, 277.4 and 330.8 cm^−1^ stem from the *A*_1g_, *E*_2g_ and *B*_2g_ modes of 2H-MoSe_2_, respectively, while the peak at 142.1 cm^−1^ is associated to the *E*_1g_ mode of the in-plane bending of Se atoms in 2H-MoSe_2_^32^. For the Raman spectra of S_v_-ZIS, the peaks located at 244.8 and 348.9 cm^−1^ can be severally assigned to the *F*_2g_ and *A*_1g_ modes of ZnIn_2_S_4_. Furtherly, as for the S_v_-ZIS/MoSe_2_ (the red line), in addition to the *E*_1g_ mode of 2H-MoSe_2_, and the *F*_2g_ and *A*_1g_ modes of ZnIn_2_S_4_, a new emerging peak situated at about 404.9 cm^−1^ can be indexed to the Mo-S bonding state^33^, suggesting that the S_v_-ZIS and MoSe_2_ were combined intimately by Mo-S bond. Additionally, it can be observed that all the peaks in S_v_-ZIS/MoSe_2_ exhibited evidently blue-shift compared to that in S_v_-ZIS, further revealing the intense chemical coupling effect between the S_v_-ZIS and MoSe_2_^34^.

To further testify the existence of S vacancies, the electron paramagnetic resonance (EPR) was carried out (Fig. 2s). For the original ZIS sample, the EPR intensity can hardly be observed, in comparison, the S_v_-ZIS sample shows the sharply increased EPR signal at a g-factor of 2.009, confirming the abundant S-vacancies in S_v_-ZIS^35,36^. In addition, it is interesting to observe that the EPR intensity of S_v_-ZIS/MoSe_2_ exhibits slightly decreased compared to that of S_v_-ZIS, which should be contributed to the bonding effect among Mo and unsaturated S in S_v_-ZIS, decreasing the number of unpaired electrons, but the S vacancies in ZIS have not been sewed up by compositing MoSe_2_^37^.

**Fig. 3:**
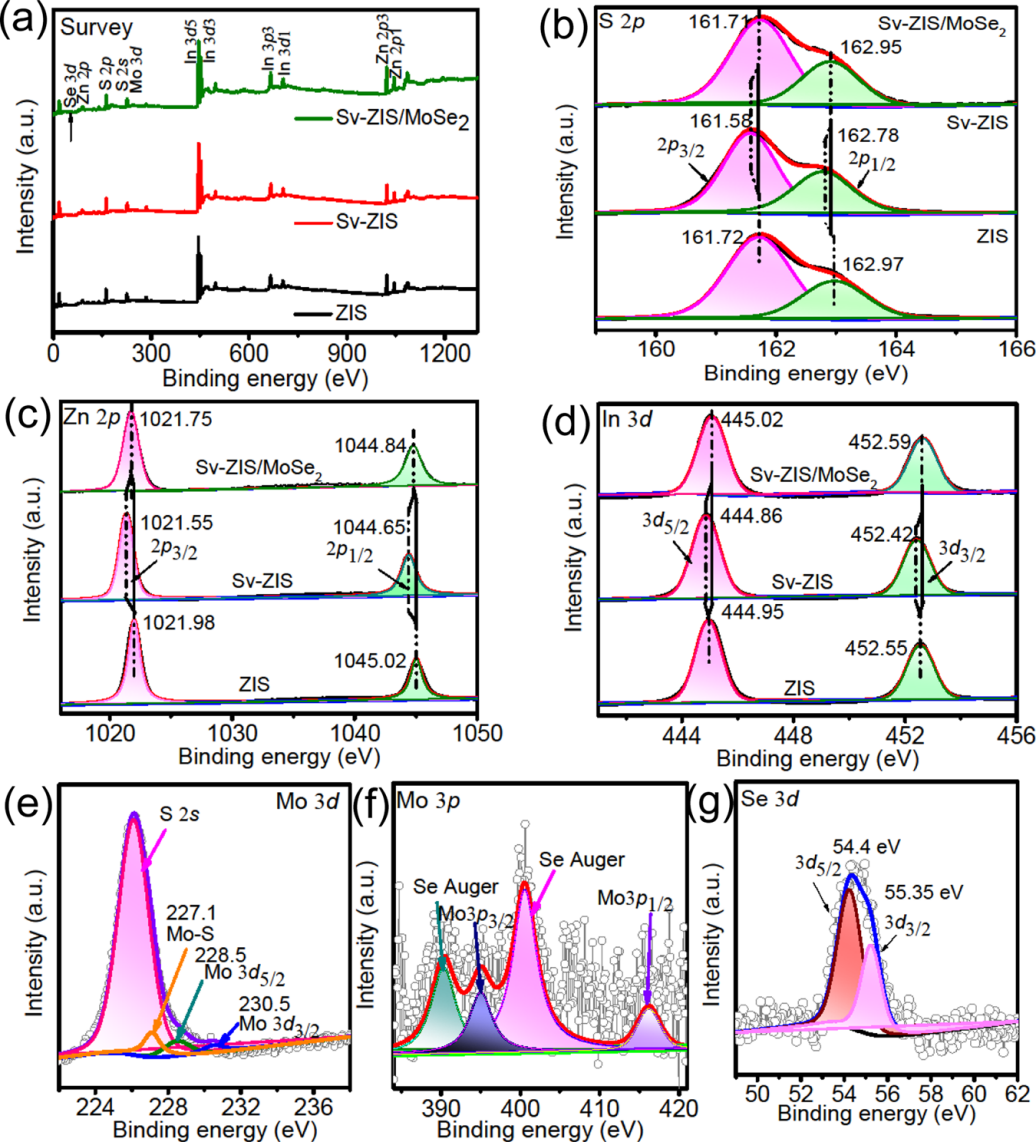
XPS spectra. **a** survey, **b** S 2*p*, **c** Zn 2*p*, **d** In 3*d* for ZIS, S_v_-ZIS and S_v_-ZIS/MoSe_2_, **e** Mo 3*d* and S 2 *s*, **f** Mo 3*p* and **g** Se 3*d* of S_v_-ZIS/MoSe_2_.

The X-ray photoelectron spectroscopy (XPS) was applied to investigate the surface composition and chemical states of ZIS, S_v_-ZIS and S_v_-ZIS/MoSe_2_, and the results are showing in Fig. 3. As can be found from the survey spectrum (Fig. 3a), the Zn, In and S peaks are coexisting in ZIS and S_v_-ZIS, in comparison, Mo and Se peaks can also be observed in the S_v_-ZIS/MoSe_2_, which is agree with the EDS test results. As observed in Fig. 3b, the S 2*p*_3/2_ and 2*p*_1/2_ of the original ZIS located at 161.72 and 162.97 eV, respectively, in accordance with the reported literature^36^. In comparison, the S 2*p*_3/2_ and 2*p*_1/2_ of S_v_-ZIS presented evident negative-shift of about 0.14 eV and 0.19 eV, respectively, verifying the generation of S vacancies in ZIS. The S-vacancies can serve as strong electron-withdrawing group for facilitating the ZIS electrons transfer to S-vacancies, thus decreasing the equilibrium electron cloud density of S atoms inside ZIS, and further leading to the decreased binding energy^38,39^. Furtherly, it can be noted that the S 2*p*_3/2_ and 2*p*_1/2_ of S_v_-ZIS/MoSe_2_ exhibited a positive-shift of about 0.13 and 0.17 eV compared to that of S_v_-ZIS, which should be caused by the strong interfacial interaction between MoSe_2_ and S_v_-ZIS^34^. Besides, as shown in Fig. 3c and d, the Zn 2*p* and In 3*d* in S_v_-ZIS also exhibited a slightly negative-shift compared to that in ZIS, which could be explained that the generation of S vacancies leading to the decreased coordination number of Zn and In^37^. After combining with MoSe_2_, the Zn 2*p* and In 3*d* peaks re-shift to the high binding energy region, revealing that the bonding effect between Mo atoms in MoSe_2_ and unsaturated coordination S in S_v_-ZIS contributing to the slightly increased electron cloud density around Zn and In. Interestingly, it can also be observed that the binding energy variation of Zn 2*p* in ZIS, S_v_-ZIS, and S_v_-ZIS/MoSe_2_ are more notable than that of In 3*d*, revealing that the Mo were mainly bonded with the S around Zn sites^37^. What’s more, according to the XPS peak area, the actual atomic ratio of Zn/In/S in ZIS, S_v_-ZIS and S_v_-ZIS/MoSe_2_ are 1.00/2.15/3.87, 1.00/2.20/3.29, and 1.00/2.14/3.36, respectively. The lower S atom ratio in S_v_-ZIS and S_v_-ZIS/MoSe_2_ further confirm the presence of abundant S vacancies. As shown in Fig. 3e, the peaks at 228.05 and 230.5 eV can be attributed to Mo 3*d*_5/2_ and 3*d*_3/2_ of Mo^4+^ in MoSe_2_, meanwhile, the peak at 227.1 eV verified the formation of Mo-S bond^40^. Figure 3f is the Mo 3*p* spectrum, as observed, four distinct XPS peaks can be distinguished, where the peaks at 400.55 and 390.3 eV can be corresponded to the Se Auger peaks, and the peaks at 395 and 416 eV can be assigned to the Mo 3*p*_3/2_ and 3*p*_1/2_ of Mo^4+^. The Se 3*d* spectrum presented in Fig. 3g shows two peaks at 54.4 and 55.35 eV, which can be indexed to Se 3*d*_5/2_ and 3*d*_3/2_ of Se^2-^ in MoSe_2_, respectively^32^. The XPS results further confirm the successful synthesis of S_v_-ZIS and S_v_-ZIS/MoSe_2_ with abundant S-vacancies, and the MoSe_2_ is attached on the surface of S_v_-ZIS through Mo-S bond.

### Photocatalytic H_2_ evolution activity measurements

**Fig. 4:**
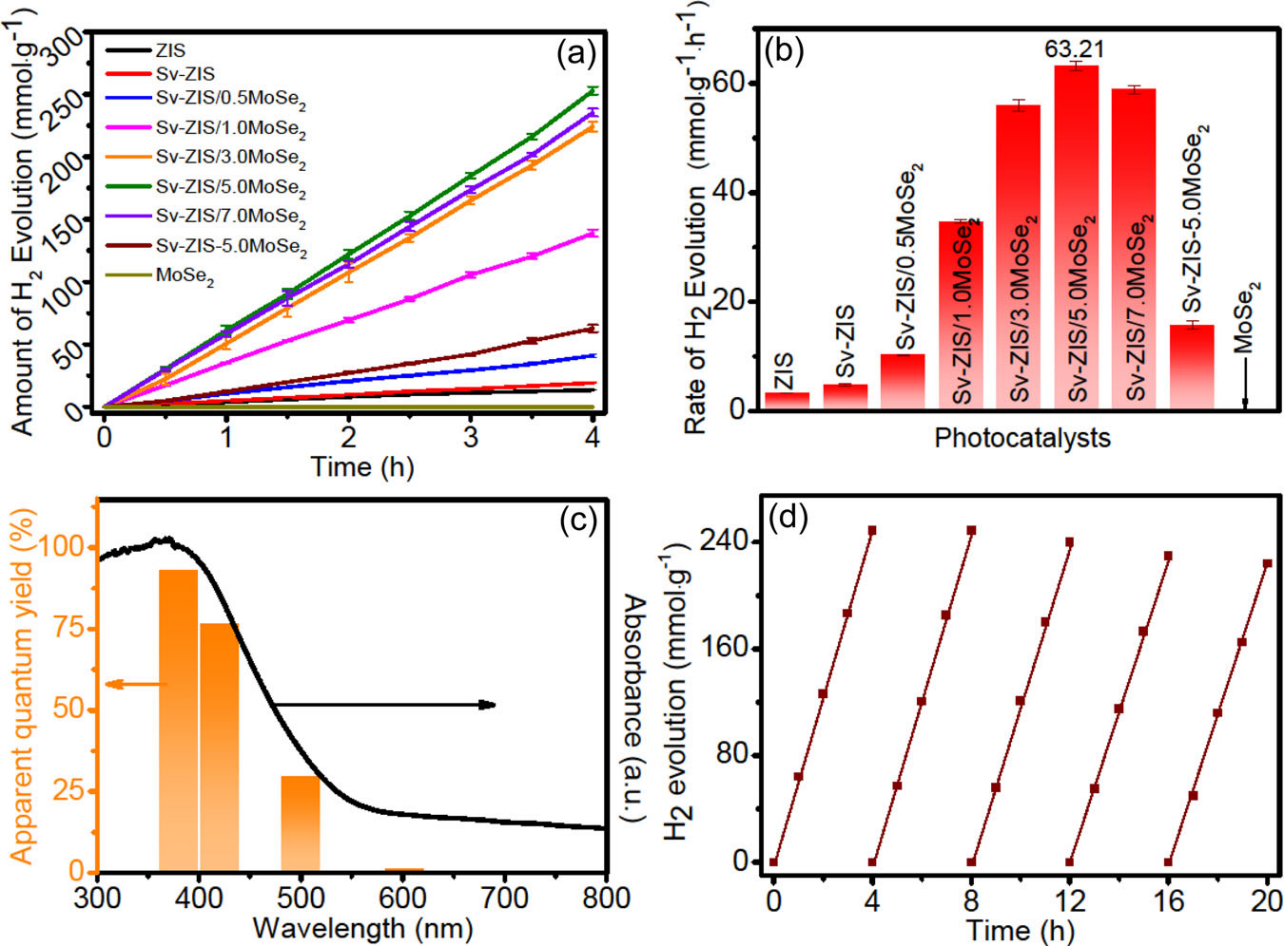
Photocatalytic H_2_ evolution property. **a** H_2_ evolution amount at different irradiation time and **b** H_2_ evolution rate of different photocatalysts, **c** wavelength-dependent apparent quantum yield (AQY) and **d** cycling stability test of S_v_-ZIS/5.0MoSe_2_. The vertical error bars indicate the maximum and minimum values obtained; the dot represents the average value.

The photocatalytic H_2_ evolution were evaluated under the visible light (λ > 420 nm) irradiation, the corresponding test results are showing in Fig. 4. As shown in Fig. 4a and b, all the tested samples exhibit H_2_ production activity except for MoSe_2_. The pristine ZIS exhibits the poor H_2_ production activity of about only 3.36 mmol∙g^−1^·h^−1^, in comparison, the S_v_-ZIS presents a slightly improved H_2_ evolution rate of 4.77 mmol∙g^−1^·h^−1^. The improved photocatalytic performance of S_v_-ZIS should be ascribed to the accelerated photocarriers separation induced by S vacancies as the electrons trap. Furtherly, the introduction of MoSe_2_ gave rise to the distinctly improved H_2_ evolution activity, and the H_2_ evolution rate of S_v_-ZIS/MoSe_2_ increased with the mass ratio of MoSe_2_ to ZIS increasing. Until the mass ratio of MoSe_2_ to ZIS reaches to 5.0%, the H_2_ evolution rate reaches to the highest of 63.21 mmol∙g^−1^·h^−1^, which is about 18.8 and 13.3 times higher than that of pristine ZIS and S_v_-ZIS, respectively, and superior to the recently reported ZnIn_2_S_4_-based photocatalytic system (as listed in Table S6). It can also be observed that the S_v_-ZIS-5.0MoSe_2_ (synthesized by mixing S_v_-ZIS and MoSe_2_ by ultrasound) performs obvious inferior H_2_ evolution property compared to that of S_v_-ZIS/5.0MoSe_2_, indicating that the in-situ growth of MoSe_2_ on S_v_-ZIS connecting by Mo-S bond plays critical influence on the photocatalytic performance of the ZIS-MoSe_2_ composite, which should be attributed to that the Mo-S bond could facilitate the charge transfer between S_v_-ZIS and MoSe_2_. Besides, Fig. S5 shows the wavelength dependent hydrogen evolution efficiency of S_v_-ZIS/MoSe_2_, which was tested following the similar procedure of photocatalytic H_2_ evolution, except that the band-pass filter was equipped to obtain monochromatic incident light (λ=380, 420, 500 and 600 nm). The detailed test results and the light power of different monochromatic light are displaying in Table S5. Accordingly, the AQY of photocatalytic H_2_ evolution over the S_v_-ZIS/MoSe_2_ photocatalyst can be calculated (the detailed calculation process is shown in the Supporting Information) and the action spectrum was displayed in Fig. 4c. As observed, the action spectrum of S_v_-ZIS/MoSe_2_ matches well with the UV-vis absorption spectra, besides, the AQY values of S_v_-ZIS/MoSe_2_ are about 93.08% (380 nm), 76.48% (420 nm), 29.7% (500 nm) and 0.15% (600 nm), indicating the favorable optical absorption and utilization capacity of S_v_-ZIS/MoSe_2_ photocatalyst. Fig. S6 is the AQY of ZIS and S_v_-ZIS, it can be observed that under different monochromatic light wavelength, the AQY of S_v_-ZIS are larger than that of ZIS, suggesting the more efficient photons to H_2_ conversion ability of S_v_-ZIS, which should be caused by the enhanced light absorption and the promoted photocarriers separation efficiency by introducing abundant S-vacancies in S_v_-ZIS. In addition to the excellent photocatalytic H_2_ evolution efficiency, the recycling stability is also a pivotal factor for the practical application of photocatalyst. As discerned in Fig. 4d, the H_2_ evolution amount of the optimized S_v_-ZIS/MoSe_2_ photocatalyst remains about 90.5% after 20 h of 5 cycles of photocatalytic tests, signifying the favorable photocatalytic stability of S_v_-ZIS/MoSe_2_ photocatalyst, which maybe contributed to the strong combination between ZIS and MoSe_2_ through Mo-S bond.

### Photophysical and Electrochemical Properties

**Fig. 5:**
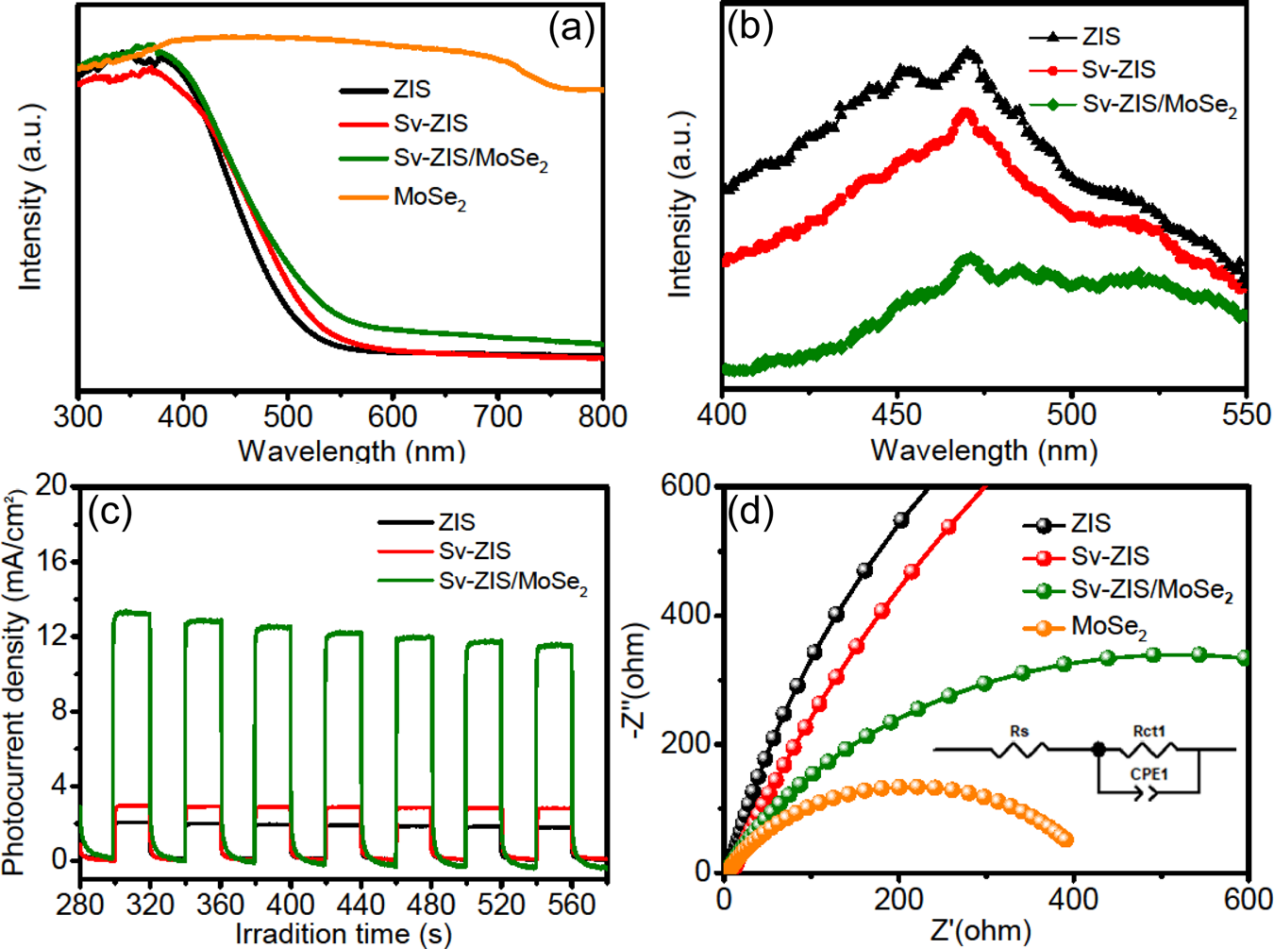
Photophysical and Electrochemical measurements. **a** UV-vis absorption spectrum, **b** photoluminescence spectra (PL, excited at 375 nm), **c** photocurrent response and **d** electrochemical impedance spectroscopy (EIS) of the as-prepared samples.

Figure 5a is the UV-vis absorption spectra of ZIS, S_v_-ZIS, MoSe_2_ and S_v_-ZIS/MoSe_2_. It is apparent that the MoSe_2_ shows the intense light absorption in the whole UV-vis light range, which should be caused by its dark black color. Meanwhile, it can be observed that light absorption intensity of S_v_-ZIS is higher than that of ZIS, indicating that the introduction of S vacancies can influence the band structure of ZIS. Furtherly, after combining with MoSe_2_, the light absorption of S_v_-ZIS/MoSe_2_ increased again compared to S_v_-ZIS. The improved light absorption is in favor of the generation of photocarriers, and beneficial for the enhancement of photocatalytic performance^9^. Figure 5b is the PL spectroscopy. As displayed, under the 375 nm excitation wavelength, the pristine ZIS displays a prominent emission peak, indicating the intense recombination of photogenerated carriers inside ZIS. In comparison to ZIS, the emission peak intensity of S_v_-ZIS decreases lightly, which should be contributed to that S vacancies can act as electrons trap for facilitating the photocarriers separation. It is worth noting that the PL signal of S_v_-ZIS/MoSe_2_ sample is further quenched compared to that of S_v_-ZIS, revealing the positive effect of MoSe_2_ for suppressing the recombination of photocarriers. Figure 5c is the photocurrent response. As observed, all the tested samples exhibit the light-response characteristic under the FX-300 Xe lamp. Obviously, the photocurrent density is in the order of S_v_-ZIS/MoSe_2_ > S_v_-ZIS > ZIS. The highest photocurrent density of S_v_-ZIS/MoSe_2_ reveals the most accelerated photocarriers separation and migration efficiency. Figure 5d is the electrochemical impedance spectroscopy (EIS). As compared, MoSe_2_ express the smallest semicircle, meanwhile, the semicircle of ZIS is the largest. Obviously, the semicircle of S_v_-ZIS is slightly lower than that of ZIS, and the semicircle of S_v_-ZIS/MoSe_2_ is significantly decreased than that of pristine ZIS and S_v_-ZIS, manifesting that the introduction of S vacancies and the combination with MoSe_2_ can decrease the interfacial charge transfer resistance, which is in favor of photogenerated carriers transfer and separation, and finally facilitate the photocatalytic property.

In order to investigate the effects of the MoSe_2_ to ZIS mass ratio on the photocatalytic performance of S_v_-ZIS/MoSe_2_ composites. The light absorption, photocarriers separation and photocurrent density of S_v_-ZIS/MoSe_2_ photocatalysts with different mass ratio of MoSe_2_ to ZIS were also characterized by UV-vis absorption, steady-state PL spectroscopy and photocurrent response. As observed in Fig. S7, with increasing the mass ratio of MoSe_2_ to ZIS, the light absorption intensity enhance gradually. It is worth mentioning that the S_v_-ZIS/7.0MoSe_2_ sample displays the strongest light absorption ability, but its photocatalytic H_2_ production performance is not the best (as known from Fig. 4a), suggesting that the light absorption is not the only decisive factor for the photocatalytic activity. Fig. S8 is the PL spectra, it can be observed that the PL peak of S_v_-ZIS/5.0MoSe_2_ is the lowermost, revealing the most effective photocarriers separation when the mass ratio of MoSe_2_ to ZIS is 5%, which directly explains why the S_v_-ZIS/5.0MoSe_2_ sample has the best photocatalytic performance. Figure S9 shows the photocurrent response. As displayed, the S_v_-ZIS/5.0MoSe_2_ shows the highest photocurrent density, which is the result of high-efficiency separation and transfer of photogenerated electron and hole, further revealing the optimum photocatalytic performance of S_v_-ZIS/5.0MoSe_2_. As known from the above results, the prominent photocatalytic performance requires the coordination among the efficient light absorption, photocarrier separation and transfer ability.

### Mechanism analysis

**Fig. 6:**
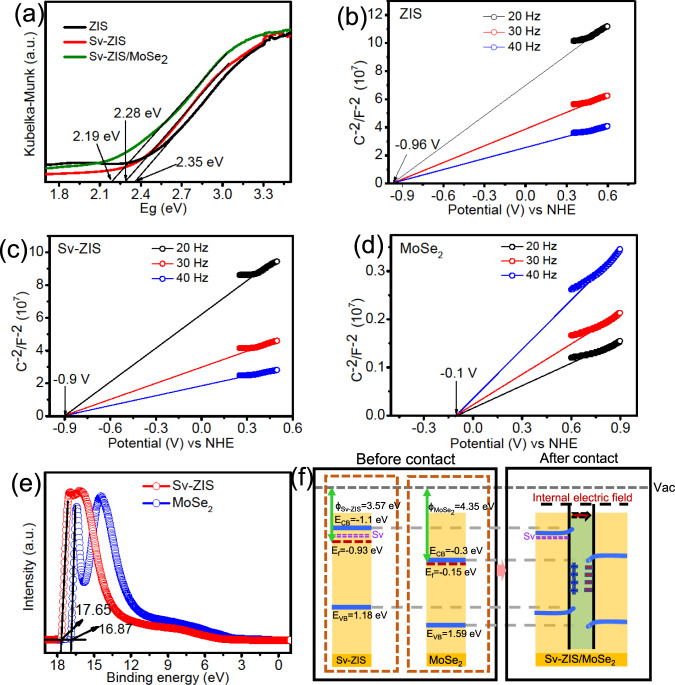
Band structure and the formation of internal electric field. **a** Kubelka-Munk function vs. the energy of incident light plots, **b–d** Mott-Schottky (M-S) plot, **e** UPS spectra of the as-prepared samples, and **f** band structure of S_v_-ZIS, MoSe_2_ and S_v_-ZIS/MoSe_2_.

Furtherly, the bandgap value (E_g_) of the tested sample can be obtained from the Kubelka-Munk function vs. the energy of incident light plots^41^. As displayed in Fig. 6a, the E_g_ of ZIS, S_v_-ZIS and S_v_-ZIS/MoSe_2_ can be estimated to be 2.35, 2.28 and 2.19 eV, respectively. The narrower E_g_ is beneficial for the incident light absorption and photocarriers generation, thereby contributing to the photocatalytic property^42^. The Mott-Schottky (M-S) plot can be obtained by the following formula of ${{C}}_{{sc}}^{-2}=\frac{2}{\varepsilon {\varepsilon }_{0}e{N}_{D}}\left(E-{E}_{{fb}}-\frac{{k}_{B}T}{e}\right)$, in which C_SC_ represents space charge capacitance, ɛ represents the dielectric constant, ɛ_0_ represents the permittivity of vacuum, e represents the single electron charge, N_D_ represents the charge carrier density, E_fb_ represents the flat band potential, k_B_ represents the Boltzmann constant, and T represents the temperature, E represents the electrode potential^9^. As displayed in Fig. 6b-d, the E_fb_ of ZIS, S_v_-ZIS and MoSe_2_ can be determined to be −0.96, −0.9 and −0.1 V (vs. NHE), respectively, by extending the linear part of M-S plots. Besides, all the tested samples exhibit the positive slope of M-S plots, indicating the n-type semiconductor traits^43^. As known, the conduction band potential (E_CB_) of n-type semiconductor is ~0.2 eV negative than the E_fb_^44^, thus the E_CB_ of ZIS, S_v_-ZIS and MoSe_2_ can be discerned to −1.16, −1.1 and −0.3 V (vs. NHE), respectively. According to the equation of E_VB_ = E_CB_ + E_g_ (E_VB_ is the potential of valence band (VB)), the E_VB_ of the ZIS and S_v_-ZIS can be estimated to 1.19 and 1.18 V vs. NHE, respectively. According to the reported literature, the E_g_ of MoSe_2_ is about 1.89 eV, therefore, the E_VB_ of MoSe_2_ can be determined to be 1.59 eV^25^.

The work function (ɸ) is an important nature for reflecting the escaping ability of free electron from Fermi level (E_f_) to vacuum level^45^. To investigate the mechanism for the excellent photocatalytic performance of S_v_-ZIS/MoSe_2_, the ultraviolet photoelectron spectroscopy (UPS) with He I as the excitation source was conducted. As displayed in Fig. 6e, the secondary cutoff binding energy (E_cutoff_) of S_v_-ZIS and MoSe_2_ can be respectively determined as 17.65 and 16.87 eV, by extrapolating the linear part to the base line of the UPS spectra. Based on the formula of ɸ=hv-E_cutoff_, the ɸ of S_v_-ZIS and MoSe_2_ can be calculated as 3.57 and 4.35 eV, respectively. Hence, the E_f_ of S_v_-ZIS and MoSe_2_ can be determined as −0.93 and −0.15 V (vs. NHE), respectively. Based on the above calculation and analysis results, the detailed band structure of S_v_-ZIS, MoSe_2_ and S_v_-ZIS/MoSe_2_ were depicted in Fig. 6f. As observed, the E_f_ of MoSe_2_ is below that of S_v_-ZIS, hence, when S_v_-ZIS and MoSe_2_ contact and form an intimate interface, the free electrons in S_v_-ZIS with high E_f_ would spontaneously diffuse to MoSe_2_ with low E_f_, until a new equilibrium state E_f_ fabricated. The electron drifting from S_v_-ZIS to MoSe_2_ result in the charge redistribution on the interface between S_v_-ZIS and MoSe_2_, in which the interface near S_v_-ZIS side is positively charged, while negatively charged near the MoSe_2_ side, as result, an internal electric field from S_v_-ZIS to MoSe_2_ was built^46^.

**Fig. 7:**
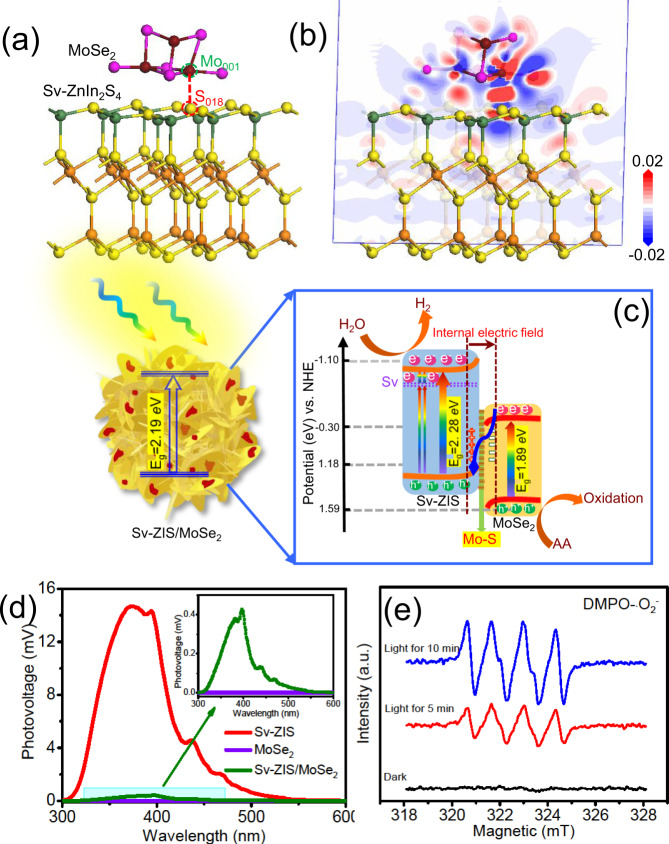
Photocatalytic mechanism and verification. **a** The optimized structure and **b** the side view of charge density difference of S_v_-ZnIn_2_S_4_/MoSe_2_ heterostructure. **c** photocatalytic reaction mechanism of S_v_-ZIS/MoSe_2_ under light irradiation, **d** Surface photovoltage (SPV) measurement of S_v_-ZIS, MoSe_2_ and S_v_-ZIS/MoSe_2_, and **e** DMPO spin-trapping electron paramagnetic resonance (EPR) spectra of DMPO- ∙ O_2_^-^ of S_v_-ZIS/MoSe_2_ in methanol solution.

To further reveal the photocatalytic reaction mechanism of S_v_-ZnIn_2_S_4_/MoSe_2_ heterostructure, the density functional theory (DFT) calculations were conducted out. Figure 7(a) is the optimized structure of S_v_-ZnIn_2_S_4_/MoSe_2_ heterostructure, where the coordinative unsaturation S atoms was simulated by breaking two Zn-S bonds in the surface of ZnIn_2_S_4_. According to Population analysis and Hirshfeld analysis results, the population of Mo_001_–S_018_ is 0.34, and the transferred charge between MoSe_2_ and S_v_-ZnIn_2_S_4_ is 0.12 | e | . The above results directly demonstrate the intense bonding effect between the Mo atom in MoSe_2_ and the coordinative unsaturation S atom in ZnIn_2_S_4_. Figure 7(b) shows the side view of charge density difference of S_v_-ZnIn_2_S_4_/MoSe_2_, where the red and blue iso-surfaces denote the accumulation and depletion of electron density, respectively. As observed, the electron cloud density presents distinctly localized distribution between the Mo atom in MoSe_2_ and the coordinative unsaturation S atoms in S_v_-ZnIn_2_S_4_, which more intuitively manifests the intense bonding effect between Mo and S. In addition, it can be noted that the surface of MoSe_2_ was dominantly covered by red color, while S_v_-ZnIn_2_S_4_ was chiefly filled by blue color, suggesting that the electrons in S_v_-ZnIn_2_S_4_ were transfer to MoSe_2_ along the intimate heterointerface, which would subsequently induce the internal electric field in S_v_-ZnIn_2_S_4_/MoSe_2_ heterostructure^47^.

Accordingly, the photocatalytic reaction mechanism of S_v_-ZIS/MoSe_2_ can be elaborated in Fig. 7c. Under the irradiation of visible light, a mass of photoinduced electrons (e^-^) with enough energy would transfer from the VB of S_v_-ZIS and MoSe_2_ to the CB of S_v_-ZIS and MoSe_2_, respectively, while the holes (h^+^) be left on the VB of S_v_-ZIS and MoSe_2_, respectively. It should be mentioned that the abundant S vacancies inside ZIS could introduce new donor level in the band gap of ZIS, which can act as efficient electrons trap to suppress the photogenerated electron-hole pairs recombination^48^. Furtherly, under the driving effect of the internal electric field, the electrons on the CB of MoSe_2_ would migrate to the VB of S_v_-ZIS to recombine with the holes. The Mo-S bond acting as atomic-level interfacial “bridge” can promote the photoexcited carriers migration between S_v_-ZIS and MoSe_2_, thus significantly accelerating the Z-scheme charge transfer. To validate the Z-scheme charge transfer mechanism, the SPV and EPR measurements were carried out. Figure 7d is the SPV spectra of S_v_-ZIS, MoSe_2_ and S_v_-ZIS/MoSe_2_ samples. It is noted that the pristine MoSe_2_ presents no SPV signals in the whole wavelength, suggesting the poor photocarriers separation efficiency inside the MoSe_2_, that’s why MoSe_2_ performed very poor hydrogen evolution. In comparison, a significant positive photovoltage response can be observed in the SPV spectra of S_v_-ZIS, suggesting that the holes migrate to the surface of S_v_-ZIS, which is the typical trait of n-type semiconductor^49^. Meanwhile, the SPV response of S_v_-ZIS/MoSe_2_ is significantly lower than that of S_v_-ZIS, which means that fewer photogenerated holes migrate to the surface of S_v_-ZIS/MoSe_2_. This phenomenon should be contributed to that the photogenerated electrons on the CB of MoSe_2_ transfer to the VB of S_v_-ZIS and recombine with the photogenerated holes, that’s the Z-scheme mechanism^50^. EPR spin-trapping experiment with DMPO as spin-trapping reagent was further proceeded to support the Z-scheme charge transfer mechanism in S_v_-ZIS/MoSe_2_. As displayed in Fig. 7e, almost no DMPO- ∙ O_2_^-^ signals can be observed under dark conditions. However, under visible light irradiation, the characteristic peaks of DMPO- ∙ O_2_^-^ (1:1:1:1) can be monitored for the S_v_-ZIS/MoSe_2_ methanol dispersion liquid, and the peak intensity increase with the time extending, suggesting that the ∙O_2_^-^ was generated in the reaction system^51^. In theory, the electrons on MoSe_2_ cannot reduce O_2_ to product ∙O_2_^-^ due to the lower CB potential of MoSe_2_ (−0.3 V vs. NHE) than the redox potential of O_2_/ ∙ O_2_^-^ (−0.33 V vs. NHE)^52^. Therefore, the ∙O_2_^-^ should be the reaction product between the photoinduced electrons on the CB of S_v_-ZIS and O_2_ (the CB potential of S_v_-ZIS is about −1.10 eV, lager than the redox potential of O_2_/ ∙ O_2_^-^), indicating that a mass of photogenerated electrons were accumulated on the CB of S_v_-ZIS under irradiation of visible light, which should be contributed by the recombination between the electron on the CB of MoSe_2_ and the hole on the VB of S_v_-ZIS, thus verifying the direct Z-scheme charge migration mechanism. Above SPV and EPR spin-trapping technique provides the direct proof for the direct Z-scheme charge transfer mechanism inside the S_v_-ZIS/MoSe_2_ photocatalyst.

In summary, we have successfully demonstrated an interfacial Mo-S bond and internal electric field modulated Z-scheme S_v_-ZnIn_2_S_4_/MoSe_2_ photocatalyst through a defect-induced heterostructure constructing strategy for boosting the photocatalytic H_2_ evolution performance. The internal electric field provide the necessary driving force steering the photogenerated electrons on the conduction band of MoSe_2_ transfer to the valence band of S_v_-ZnIn_2_S_4_ following the Z-scheme mechanism, while the interfacial Mo-S bond creates direct charge transfer channels between S_v_-ZnIn_2_S_4_ and MoSe_2_, further accelerates the Z-scheme charge transfer process. What’s more, the abundant S-vacancies also contribute to the enhanced light absorption and accelerated photocarriers separation. The above factors together lead to the efficient photocatalytic performance of the S_v_-ZnIn_2_S_4_/MoSe_2_. Specifically, the optimized photocatalyst exhibits a high AQY of 76.48% at 420 nm, and an ultrahigh H_2_ evolution rate of 63.21 mmol·g^−1^ ∙ h^−1^ under visible light (λ > 420 nm), which is about 18.8 times higher than that of pristine ZnIn_2_S_4_. Besides, the S_v_-ZnIn_2_S_4_/MoSe_2_ also shows favorable recycling stability by remaining above 90% rate retention after 20 h of 5 continuous photocatalytic tests. This work not only provides an efficient direct Z-scheme ZnIn_2_S_4_-based heterostructure photocatalyst, but also affords a beneficial prototype for designing other Z-scheme photocatalyst for efficient green energy conversion.

## Methods

### Materials

Analytical grade reagents were used directly without purification. Zinc acetate dihydrate (Zn(CH_3_COO)_2_·2H_2_O) was bought from Tianjin guangcheng chemical reagent Co. LTD. Thioacetamide (TAA), Indium chloride (InCl_3_), and Selenium power (Se, ≥99.99% metal basis) were bought from Shanghai Macklin biochemical technology Co. LTD. Ascorbic acid (AA) and hydrazine monohydrate (N_2_H_4_·H_2_O, 85%) were bought from Sinopharm Chemical Reagent Co., LTD. Sodium molybdate dihydrate (Na_2_MoO_4_·2H_2_O) was purchased from Tianjin Fengchuan Chemical Reagent Technology Co., LTD. Deionized water was obtained from local sources.

### Synthesis of ZnIn_2_S_4_ and S_v_-ZnIn_2_S_4_

In a representative experiment, InCl_3_ (1 mmol), Zn(CH_3_COO)_2_·2H_2_O (0.5 mmol), and TAA (4 mmol) were orderly dissolved into 50 mL deionized water, and then stirred at room temperature for 30 min. Thereafter, the clear solution was poured into 100 mL stainless steel autoclave, and maintained at 180 °C oven for 18 h. After cooling naturally to indoor temperature, the sediment was separated by centrifugation, followed by washing with deionized water and ethanol, and drying at 60 °C for 10 h. The obtained yellow powder ZnIn_2_S_4_ were labeled as ZIS. S_v_-ZnIn_2_S_4_ was prepared via a N_2_H_4_·H_2_O-assisted hydrothermal method. Typically, 100 mg the as-synthesized ZIS was dispersed into 20 mL deionized water for 1 h, then, 5 mL N_2_H_4_·H_2_O was added into the mixing solution and stirred for another 30 min. After that, the mixture was transfer to 50 mL stainless steel autoclave, and maintained at 240 °C oven for 5 h. Finally, the precipitate was separated by centrifugation, and washing with deionized water for several times, then drying at 60 °C for 10 h. The obtained light-yellow powder was labeled as S_v_-ZIS.

### Synthesis of S_v_-ZnIn_2_S_4_/MoSe_2_ heterostructure

The S_v_-ZnIn_2_S_4_/MoSe_2_ heterostructure were synthesized by the similar process with S_v_-ZnIn_2_S_4_, except that Na_2_MoO_4_·2H_2_O and Se powders were added into the mixture. The S_v_-ZnIn_2_S_4_/MoSe_2_ with different mass ratio of MoSe_2_ to ZnIn_2_S_4_ (0.5%, 1.0%, 3.0%, 5.0%, and 7.0%) were synthesized by adjusting the addition of Na_2_MoO_4_·2H_2_O and Se, and the synthesized samples were labeled as S_v_-ZIS/0.5MoSe_2_, S_v_-ZIS/1.0MoSe_2_, S_v_-ZIS/3.0MoSe_2_, S_v_-ZIS/5.0MoSe_2_, S_v_-ZIS/7.0MoSe_2_, respectively. For comparison, the pure MoSe_2_ was prepared following the above steps without adding ZIS. Besides, the S_v_-ZIS-5.0MoSe_2_ mixture was also fabricated by ultrasonic mixing the S_v_-ZIS with MoSe_2_ for 1 h.

### Characterization

The morphology and microstructure were investigated by SU8010 scanning electron microscope (SEM) outfitted with an energy dispersive X-ray spectrometer (EDS), and JEM-2100 plus transmission electron microscope (TEM). The crystalline and phase information were characterized by Bruker D8 Advance X-ray diffraction (XRD). The chemical states were investigated by Thermo ESCALAB 250 XI X X-ray photoelectron spectroscopy (XPS, monochromatic Al Kα radiation), and the XPS data was calibrated by C 1 s spectrum (binding energy is 284.8 eV). The light absorption property was researched by the PerkinElmer Lambda 750 S UV-vis spectrophotometer using barium sulfate as standard reference. The recombination of photogenerated carriers was tested by F-4600 spectrofluorometer (375 nm excitation wavelength). The secondary cutoff binding energy was measured by AXIS SUPRA X-ray photoelectron spectroscopy with He I as the excitation source. The surface photovoltage (SPV) measurement were carried out on the system consisting a 500 W Xe lamp source equipped with a monochromator, a lock-in amplifier with a light chopper, a photovoltaic cell, and a computer. The Raman spectra were conducted on LabRAM HR Evolution Raman spectrometer with 325 nm excitation wavelength to analysis the composition. The electron paramagnetic resonance (EPR) measurement was conducted on JEOL JES-FA200 EPR spectrometer with a 9.054 GHz magnetic field. The 5,5-dimethyl-pyrroline N-oxide (DMPO) was adopted as spin-trapping reagent and the ∙O_2_^-^ and ∙OH were tested in methanol and aqueous solution, respectively.

### Photocatalytic water splitting for hydrogen evolution

The hydrogen production experiments were proceeded on Labsolar-6A (Beijing Perfectlight). Typically, photocatalyst (50 mg) was ultrasonically suspended into 100 mL solution involving 0.1 M ascorbic acid sacrificial agent. Prior to exerting light, the reaction system was degassed for 1 h to thoroughly exclude the air and the dissolved oxygen in reaction system. Then the reaction was proceeded under PLS-SEX300D 300 W Xenon lamp (Beijing Perfectlight) with a 420 nm cut-off filter. The light intensity was determined by PLMW2000 photoradiometer (Beijing Perfectlight) to be about 254 mW/cm^2^. The generated hydrogen was analyzed by GC 7900 gas chromatograph (Techcomp, 5 Å molecular sieve stainless steel packed column, Ar as carrier gas and TCD detector).

### Photoelectrochemical and electrochemical measurements

All the electrochemical and photoelectrochemical measurements were conducted by a three-electrode system on CHI-660E electrochemical workstation. In the typical three-electrode system, the working electrode was a piece of nickel foam coating with the as-prepared photocatalyst, the reference electrode was Hg/HgO, while the counter electrode was Pt wire. The electrolyte was 0.5 M Na_2_SO_4_ aqueous solution. The electrochemical impedance spectroscopy (EIS) was conducted under open-circuit potential with 0.01 to 1×10^5^ Hz frequency range and 0.005 V AC amplitude. The photocurrent response was tested under FX-300 Xe lamp. Mott-Schottky (M-S) plots were collected from −1 to −0.2 V under 10 kHz frequency and 0.01 V amplitude.

The working electrode was fabricated as follows: a certain amount of photocatalyst, carbon black and polyvinylidene fluoride were weighted according to the mass ratio of 8:1:1, and then dispersed into N-methyl-2-pyrrolidone to gain a homogeneous paste. The paste was daubed on a piece of pre-cleaned 1×1 cm^2^ FTO collector, and then dried in 60 °C vacuum for 1 h.

### Theoretical calculation

Density functional theory (DFT) calculations were performed utilizing the CASTEP module of Materials Studio 6.1^53^, the Perdew-Burke-Emzerhof (PBE) functional^54^, and ultrasoft pseudopotential (USPP) method^55,56^. The cut-off kinetic energy of 400 eV, a 3×3×3 Monkhorst-pack k-point (Γ point) mesh sampled the Brillouin zone with a smearing broadening of 0.05 eV were applied during the whole process. The convergence criteria of self-consistent field (SCF), total energy difference, maximum force, and maximum displacement are 2.0×10^−6^ eV/atom, 2.0×10^−5^ eV/atom, 5.0×10^−2^ eV/Å, and 2.0×10^−3^ Å, respectively.

## Supplementary information

Supporting Information

Peer Review File

## Data Availability

The experimental data that support the findings of this study are available from the corresponding author upon reasonable request. Source data are provided with this paper.

## Source data

source data
