# Supplementary material for: Interfacial chemical bond and internal electric field modulated Z-scheme Sv-ZnIn2S4/MoSe2 photocatalyst for efficient hydrogen evolution
Source: Nat Commun. 2021 Jul 5;12:4112. doi: 10.1038/s41467-021-24511-z (PMC8257585; doi:10.1038/s41467-021-24511-z)
Supplement: Supplementary file 1 — Supporting Information [file 41467_2021_24511_MOESM1_ESM.pdf]

Supporting Information

Interfacial Chemical Bond and Internal Electric Field Modulated Z-Scheme S_v-$ZnIn_2S_4$/$MoSe_2$ Photocatalyst for Efficient Hydrogen Evolution

Xuehua Wang[1], Xianghu Wang[2], Jianfeng Huang[4], Shaoxiang Li[5], Alan Meng[2,*], Zhenjiang Li[1,3,5*]

[1] *College of Materials Science and Engineering, Qingdao University of Science and Technology, Qingdao 266042, Shandong, P. R. China*

[2] *Key Laboratory of Optic-electric Sensing and Analytical Chemistry for Life Science, MOE. College of Chemistry and Molecular Engineering, Qingdao University of Science and Technology, Qingdao 266042, Shandong, P. R. China.*

[3] *College of Sino-German Science and Technology, Qingdao University of Science and Technology, Qingdao 266061, Shandong, P. R. China.*

[4] *School of Material Science and Engineering, International S&T Cooperation Foundation of Shaanxi Province, Xi'an Key Laboratory of Green Manufacture of Ceramic Materials, Shaanxi University of Science and Technology, Xi'an 710021, China*

[5] *Shandong Engineering Technology Research Center for Advanced Coating, Qingdao University of Science and Technology, Qingdao, 266042, P. R. China*

[*] Corresponding author.

E-mail address:

zhenjiangli@qust.edu.cn (Z. J. Li); alanmengqust@163.com (A. L. Meng)

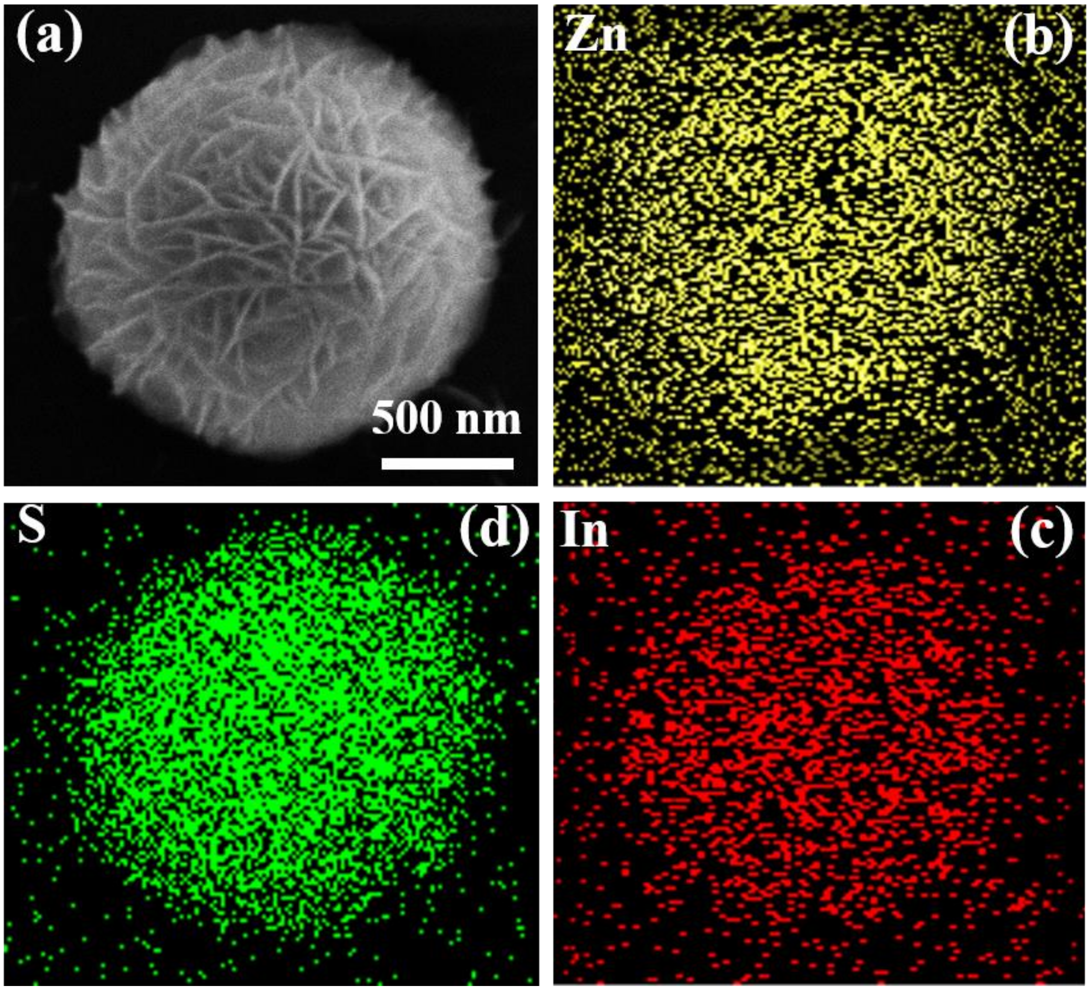

Fig. S1. Morphology characterizations of ZIS. a SEM, b-d the corresponding element mapping images of b Zn, c In and d S in ZIS

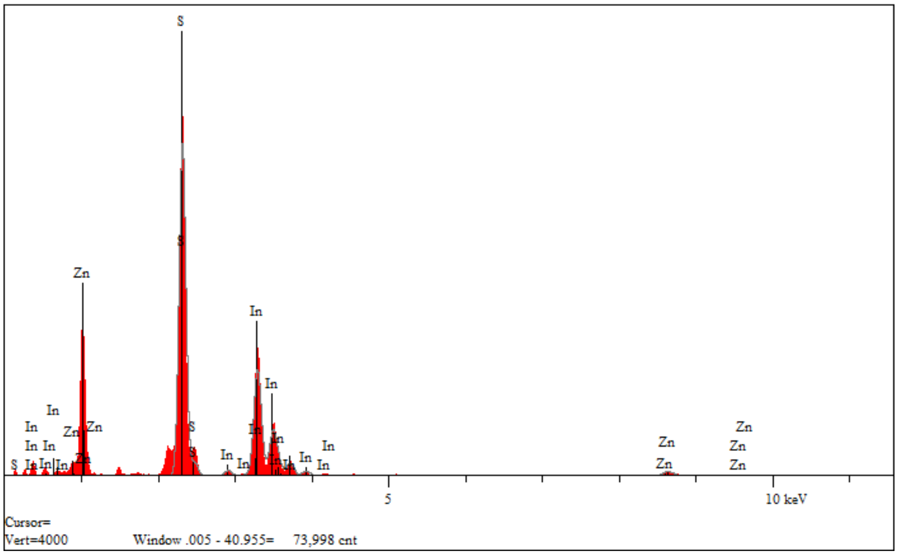

Fig. S2. EDS spectrum of ZIS.

Table S1. Elemental analyses of the ZIS photocatalyst by EDS.

Element	Atomic (%)
Zn	14.33
In	26.49
S	59.18
$Zn_{1.00}In_{1.85}S_{4.13}$	

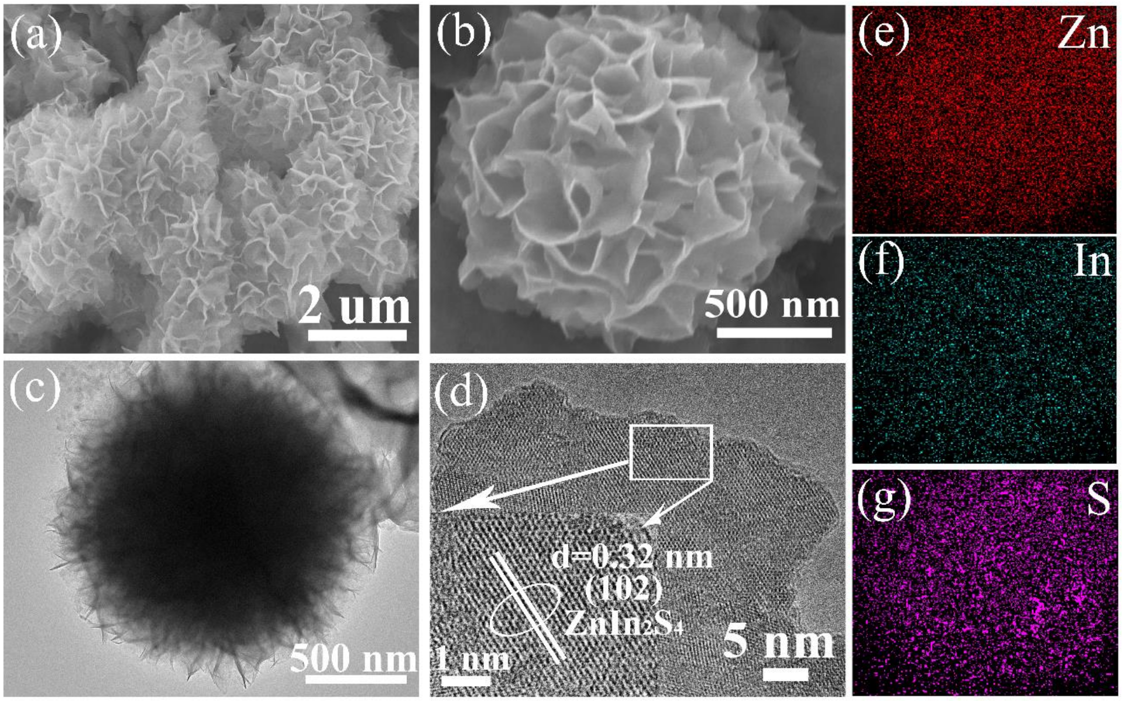

Fig.S3. Morphology characterizations of S_V-ZIS. a, b SEM, c, d TEM and HRTEM, e-g the corresponding element mapping images of c Zn, d In and e S.

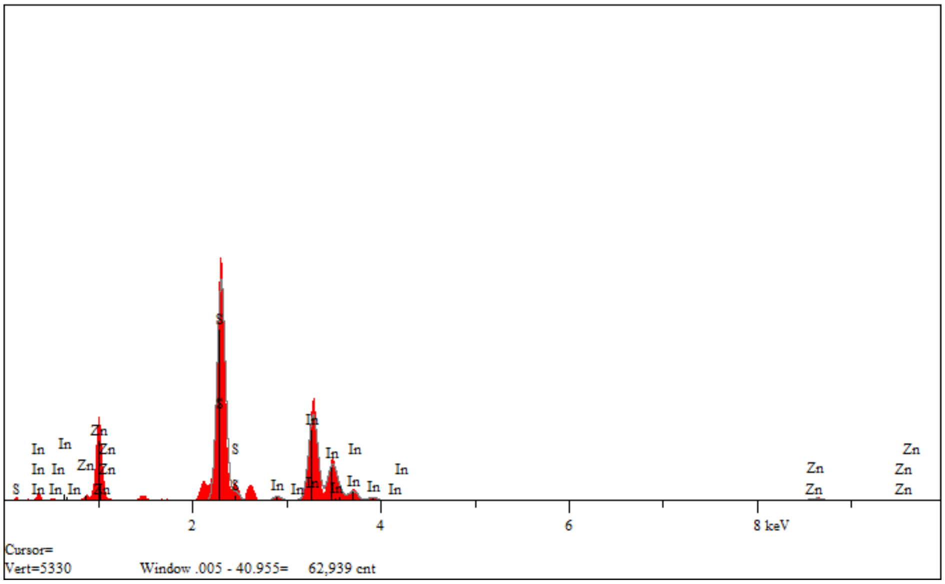

Fig. S4. EDS spectrum of S_V-ZIS.

Table S2. Elemental analyses of the S_v-ZIS photocatalyst by EDS.

Element	Atomic (%)
Zn	15.95
In	30.62
S	53.43
$Zn_{1.00}In_{1.92}S_{3.35}$	

Table S3. Elemental analyses of the S_v-ZIS/$MoSe_2$ photocatalyst by EDS.

Element	Weight (%)	Atomic (%)
Mo	2.08	1.37
Se	2.54	2.04
Zn	16.43	15.89
In	52.81	29.06
S	26.14	51.64
$Zn_{1.00}In_{1.83}S_{3.25}$		

Table S4. Elemental analyses of the S_v-ZIS/$MoSe_2$ photocatalyst by XPS.

Theoretical chemical formula	ZIS			S_v-ZIS			S_v-ZIS/$MoSe_2$		
Element	Zn	In	S	Zn	In	S	Zn	In	S
Peak area ratio	0.235	0.656	0.109	0.235	0.673	0.092	0.235	0.668	0.097
Atomic ratio	0.042	0.090	0.163	0.042	0.093	0.138	0.042	0.090	0.141
Actual chemical formula	$Zn_{1.00}In_{2.15}S_{3.87}$			$Zn_{1.00}In_{2.20}S_{3.29}$			$Zn_{1.00}In_{2.14}S_{3.36}$		

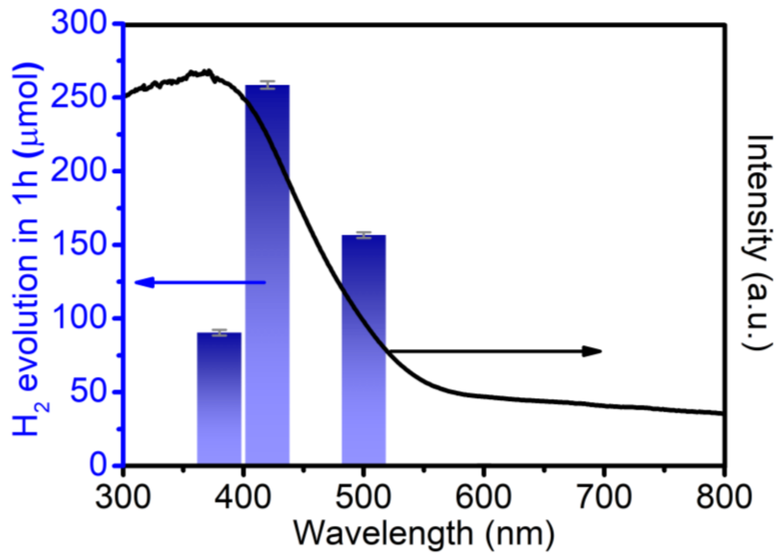

Fig. S5. Wavelength-dependent H_2 production rate in the presence of S_v-ZIS/5.0MoSe$_2$.

Table S5. The calculated AQY of S_v-ZIS/MoSe$_2$ photocatalyst at different wavelengths.

Wavelength	The mean H_2 production in 1 hour	Light power (Xe lamp)
380 nm	90.46 μmol	0.034 W
420 nm	258.53 μmol	0.107 W
500 nm	156.66 μmol	0.140 W
600 nm	0.88 μmol	0.134 W

The apparent quantum yield (AQY) was measured following the same reaction conditions as photocatalytic test except that the incident light was supplied by a 300W Xe lamp equipped with specific band-pass filters to get the desired monochromatic incident wavelength (λ=380, 420, 500 and 600 nm). AQY was roughly calculated based on the following equation:

$$AQY = \frac{the\ number\ of\ reacted\ electrons}{the\ number\ of\ incident\ photons} \times 100\%$$

$$= \frac{4 \times the\ number\ of\ H_2\ molecules}{the\ number\ of\ incident\ photons} \times 100\%$$

$$= \frac{4 \times the\ \ number\ of\ H_2\ molecules}{N} \times 100\%$$

λ=380 nm:

$$N = \frac{E\lambda}{hc} = \frac{0.034 \times 3600 \times 380 \times 10^{-9}}{6.626 \times 10^{-34} \times 3 \times 10^{8}} = 2.34 \times 10^{20}$$

$$AQY = \frac{4 \times 90.46 \times 10^{-6} \times 6.02 \times 10^{23}}{2.34 \times 10^{20}} \times 100\% = 93.08\%$$

λ=420 nm:

$$N = \frac{E\lambda}{hc} = \frac{0.107 \times 3600 \times 420 \times 10^{-9}}{6.626 \times 10^{-34} \times 3 \times 10^{8}} = 8.14 \times 10^{20}$$

$$AQY = \frac{4 \times 258.53 \times 10^{-6} \times 6.02 \times 10^{23}}{8.14 \times 10^{20}} \times 100\% = 76.48\%$$

λ=500 nm:

$$N = \frac{E\lambda}{hc} = \frac{0.140 \times 3600 \times 500 \times 10^{-9}}{6.626 \times 10^{-34} \times 3 \times 10^{8}} = 1.46 \times 10^{21}$$

$$AQY = \frac{4 \times 156.66 \times 10^{-6} \times 6.02 \times 10^{23}}{1.27 \times 10^{21}} \times 100\% = 29.70\%$$

λ=600 nm:

$$N = \frac{E\lambda}{hc} = \frac{0.134 \times 3600 \times 600 \times 10^{-9}}{6.626 \times 10^{-34} \times 3 \times 10^{8}} = 1.46 \times 10^{21}$$

$$AQY = \frac{4 \times 0.88 \times 10^{-6} \times 6.02 \times 10^{23}}{1.46 \times 10^{21}} \times 100\% = 0.15\%$$

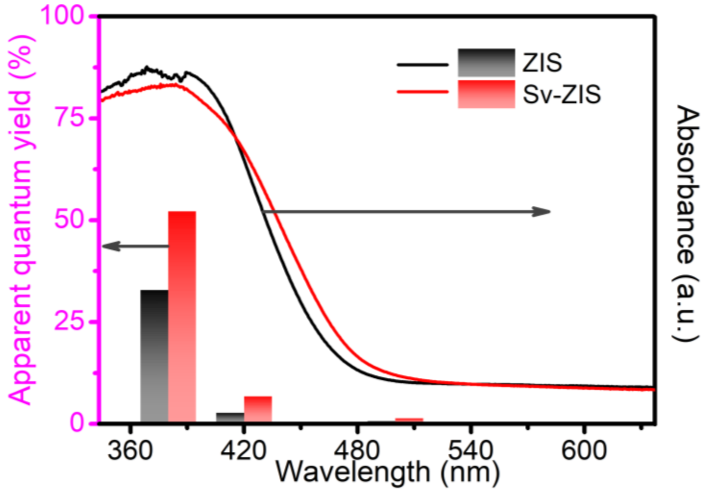

Fig. S6. Wavelength-dependent AQY for photocatalytic H2 evolution of ZIS and Sv-ZIS.

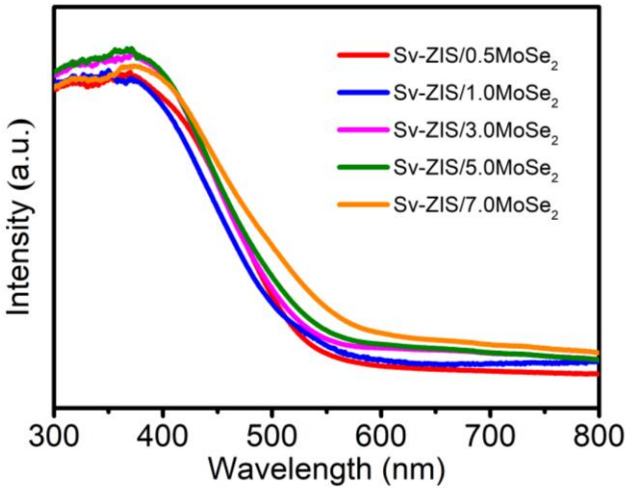

Fig. S7. UV–vis absorption spectra of S_v-ZIS/$MoSe_2$ with different mass ratio of $MoSe_2$ to ZIS.

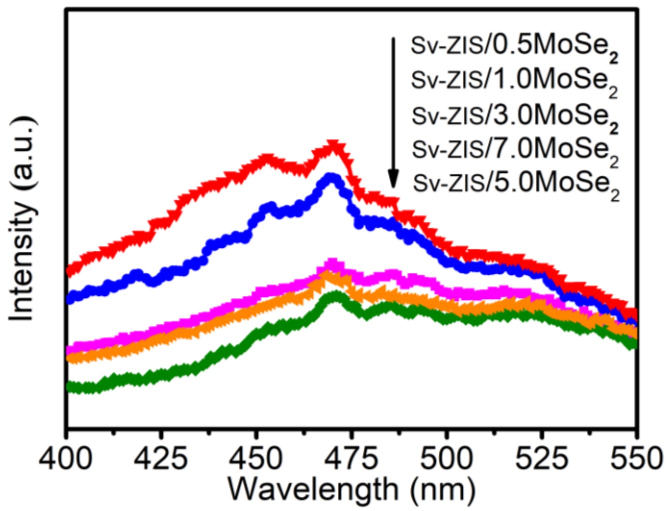

Fig. S8. Photoluminescence spectra (PL, excited at 375 nm) of S_v-ZIS/$MoSe_2$ with different mass ratio of $MoSe_2$ to ZIS.

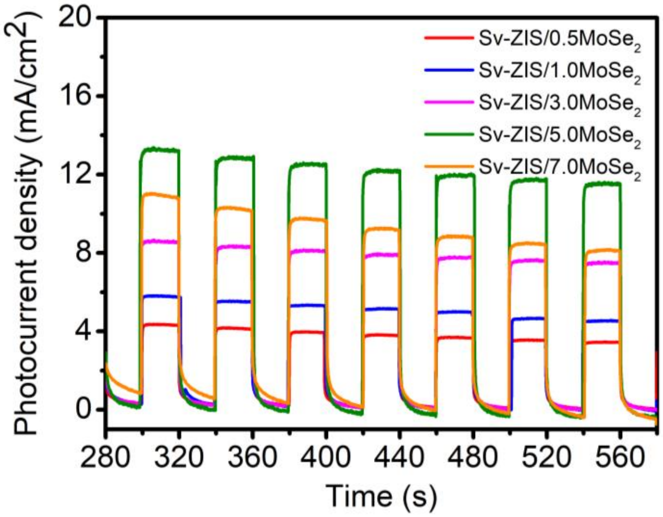

Fig. S9. Photocurrent response of S_v-ZIS/$MoSe_2$ with different mass ratio of $MoSe_2$ to ZIS.

Table S6. Comparison of representative $ZnIn_2S_4$-based photocatalysts for their H_2 evolution behavior recently.

Photocatalysts	Light source	H_2 evolution rate ($mmol \cdot g^{-1} \cdot h^{-1}$)	AQE	Refs
**$Co_9S_8/ZnIn_2S_4$**	300 W Xe lamp (λ>400 nm)	9.039	4.49% at 420 nm	1
**Co_9S_8@$ZnIn_2S_4$**	300 W Xe lamp, λ>400 nm	6.250	/	2
**$ZnIn_2S_4$-$Ti_3C_2T_X$ (3 wt% Pt co-catalyst)**	300 W Xe lamp, λ>420 nm	3.475	11.14 at 420 nm	3
**$Zn_2In_2S_5/Ti_3C_2$(O, OH) x (2 wt% Pt co-catalyst)**	300 W Xe lamp, λ>420 nm	2.596	8.96% at 420 nm	4
**g-C_3N_4/Aminated-$ZnIn_2S_4$ (1 wt% Pt co-catalyst)**	300 W Xe lamp	4.851	/	5
**N-doped $ZnIn_2S_4$**	300 W Xe lamp, λ>400 nm	11.086	16.6% at 420 nm	6
**UiO-66-$(COOH)_2/ZnIn_2S_4/MoS_2$**	300 W Xe lamp	18.794	/	7
**AgxAu1-x/$ZnIn_2S_4$**	300 W Xe lamp, λ>420 nm	5.401	8.06% at 420 nm	8
**$Zn_3In_2S_6$/FCN (2 wt% Pt co-catalyst)**	300 W Xe lamp, λ>420 nm	0.510	/	9
**$ZnIn_2S_4$/1%$MoSe_2$**	300 W Xe lamp, λ>400 nm	6.454	/	10
**Co/NGC@$ZnIn_2S_4$**	300 W Xe lamp, λ>400 nm	11.270	5.07% at 420 nm	11
**NiS/Vs-$ZnIn_2S_4/WO_3$**	300 W Xe lamp, λ>400 nm	11.090	72% at 420 nm	12
**ultra-thin $ZnIn_2S_4$/protonated g-C_3N_4**	300 W Xe lamp, λ>400 nm	8.601	0.92% at 400 nm	13
**$Ni_2P/ZnIn_2S_4$**	300 W Xe lamp, λ>420 nm	2.066	7.7% at 420 nm	14
**$ZnIn_2S_4$@NH_2-MIL-125(Ti)**	300 W Xe lamp, λ>420 nm	2.204	4.3% at 420 nm	15

**Cubic/hexagonal $ZnIn_2S_4$ heterophase junctions**	300 W Xe lamp, λ>420 nm	3.806	18.67% at 420 nm	[16]
**$ZnIn_2S_4/MoSe_2$**	300 W Xe lamp, λ>420 nm	2.228	21.39% at 420 nm	[17]
**2D/2D g-C_3N_4 nanosheet@$ZnIn_2S_4$**	300 W Xe lamp, λ>420 nm	2.780	/	[18]
**Hydrogenated $ZnIn_2S_4$**	300 W Xe lamp, λ>320 nm	0.215	13.2% at 420 nm	[19]
**$MoS_2/ZnIn_2S_4$**	300 W Xe lamp, λ>400 nm	8.898	/	[20]
**Half-unit-cell $ZnIn_2S_4$**	300 W Xe lamp, λ>400 nm	13.478	53.68% at 365 nm	[21]
**$Zn_mIn_2S_{m+3}$@In_2S_3**	300 W Xe lamp, λ>420 nm	3.300	/	[22]
**S_v-$ZnIn_2S_4/MoSe_2$**	**300 W Xe lamp, λ>420 nm**	**63.21**	**76.48% at 420 nm**	**This work**

References

1. Zhang, G. et al. Construction of hierarchical hollow $Co_9S_8/ZnIn_2S_4$ tubular heterostructures for highly efficient solar energy conversion and environmental remediation. *Angew. Chem. Int. Ed.* **59**, 8255-8261 (2020).

2. Wang, S. Guan, B. Wang, X. & Lou, X. Formation of hierarchical $Co_9S_8@ZnIn_2S_4$ heterostructured cages as an efficient photocatalyst for hydrogen evolution. *J. Am. Chem. Soc.* **140**, 15145-15148 (2018).

3. Zuo, G. et al. Ultrathin $ZnIn_2S_4$ nanosheets anchored on $Ti_3C_2T_X$ MXene for photocatalytic H_2 evolution. *Angew. Chem. Int. Ed.* **59**, 11287-11292 (2020).

4. Wang, H. et al. Electrical promotion of spatially photoinduced charge separation via interfacial-built-in quasi-alloying effect in hierarchical $Zn_2In_2S_5/Ti_3C_2(O,OH)_x$ hybrids toward efficient photocatalytic hydrogen evolution and environmental remediation. *Appl. Catal. B: Environ.* **245**, 290-301 (2019).

5. Gao, Z. et al. Aminated flower-like $ZnIn_2S_4$ coupled with benzoic acid modified g-C_3N_4 nanosheets via covalent bonds for ameliorated photocatalytic hydrogen generation. *Appl. Catal. B: Environ.* **268**, 118462 (2020).

6. Du, C. Yan, B. Lin, Z. & Yang, G. Enhanced carrier separation and increased electron density in 2D heavily N-doped $ZnIn_2S_4$ for photocatalytic hydrogen production. *J. Mater. Chem. A.* **8**, 207-217 (2020).

7. Mu, F. et al. Construction of 3D hierarchical microarchitectures of Z-scheme UiO-66-$(COOH)_2/ZnIn_2S_4$ hybrid decorated with non-noble MoS_2 cocatalyst: A highly efficient photocatalyst for hydrogen evolution and Cr(VI) reduction. *Chem. Eng. J.* **384**, 123352 (2020).

8. An, H. Li, M. Liu, R. Gao, Z. & Yin, Z. Design of Ag_xAu_{1-x} alloy/$ZnIn_2S_4$ system with tunable spectral response and Schottky barrier height for visible-light-driven hydrogen evolution. *Chem. Eng. J.* **382**, 122953 (2020).

9. Wu, Y. et al. Construction of hierarchical 2D-2D $Zn_3In_2S_6$/fluorinated polymeric carbon nitride nanosheets photocatalyst for boosting photocatalytic degradation and hydrogen production performance. *Appl. Catal. B: Environ*. **233**, 58-69 (2018).

10. Yang, M. et al. Self-surface charge exfoliation and electrostatically coordinated 2D hetero-layered hybrids. *Nat. Commun.* **8**, 14224 (2017).

11. Wang, S. et al . Supporting ultrathin $ZnIn_2S_4$ nanosheets on Co/N-doped graphitic carbon nanocages for efficient photocatalytic H_2 generation. *Adv. Mater*. **31**, 1903404 (2019).

12. Li, Z. et al. Two-dimensional Janus heterostructures for superior Z-scheme photocatalytic water splitting. *Nano Energy*. **59**, 537-544 (2019).

13. Yang, H. et al. Constructing electrostatic self-assembled 2D/2D ultra-thin $ZnIn_2S_4$/protonated g-C_3N_4 heterojunctions for excellent photocatalytic performance under visible light. *Appl. Catal. B: Environ*. **256**, 117862 (2019).

14. Li, X. et al. Fabrication of two-dimensional Ni_2P/$ZnIn_2S_4$ heterostructures for enhanced photocatalytic hydrogen evolution. *Chem. Eng. J.* **353**, 15-24 (2018).

15. Liu, H. Zhang, J. & Ao, D. Construction of heterostructured $ZnIn_2S_4$@NH_2-MIL-125(Ti) nanocomposites for visible-light-driven H_2 production. *Appl. Catal. B: Environ*. **221**, 433-442 (2018).

16. Wang, J. et al. Cubic quantum dot/hexagonal microsphere $ZnIn_2S_4$ heterophase junctions for exceptional visible-light-driven photocatalytic H_2 evolution. *J. Mater. Chem. A*. **5**, 8451-8460 (2017).

17. Zeng, D. et al. Hierarchical $ZnIn_2S_4$ /$MoSe_2$ nanoarchitectures for efficient noble-metal-free photocatalytic hydrogen evolution under visible light. *ChemSusChem*, **10**, 4624-4631 (2017).

18. Lin, B. et al. Preparation of 2D/2D g-C_3N_4 nanosheet@$ZnIn_2S_4$ nanoleaf heterojunctions with well-designed high-speed charge transfer nanochannels towards high-efficiency photocatalytic hydrogen evolution. *Appl. Catal. B: Environ.* **220**, 542-552 (2018).

19. Zhu, Y. Wang, L. Liu, Y. Shao, L. & Xia, X. In-situ hydrogenation engineering of $ZnIn_2S_4$ for promoted visible-light water splitting. *Appl. Catal. B: Environ*. **241**, 483-490 (2019).

20. Li, W. Lin, Z. & Yang, G. A 2D self-assembled MoS_2/$ZnIn_2S_4$ heterostructure for efficient photocatalytic hydrogen evolution. *Nanoscale*. 9, 18290-18298 (2017).

21. Du, C. et al. Half-unit-cell $ZnIn_2S_4$ monolayer with sulfur vacancies for photocatalytic hydrogen evolution. *Appl. Catal. B: Environ.* **48**, 193-201 (2019).

22. Li, Y. Han, P. Hou, Y. Peng, S. & Kuang, X. Oriented $ZnmIn_2S_{m+3}$@In_2S_3 heterojunction with hierarchical structure for efficient photocatalytic hydrogen evolution. *Appl. Catal. B: Environ.* **244**, 604-611 (2019).
